# Usutu, West Nile, and Tick-Borne Encephalitis Viruses

**DOI:** 10.3390/v14102120

**Published:** 2022-09-25

**Authors:** Yannick Simonin

**Affiliations:** Pathogenesis and Control of Chronic and Emerging Infections, University of Montpellier, INSERM, EFS, 34 000 Montpellier, France; yannick.simonin@umontpellier.fr

The beginning decades of the 21st century have been marked by multiple emergence and re-emergence phenomena of viral diseases. Climate change and changes in ecosystems due to biodiversity loss and modifications in land use pose environmental threats to human and animal health [1]. The emergence of infectious diseases, especially vector-borne diseases, is closely linked to modifications in ecological processes arising from those anthropogenic factors [2]. Although arboviral risk zones have already been well-identified in particular regions in Africa and Latin America, the overall trend now suggests an increase in arboviral threats in both Europe and throughout the world. Several factors may explain this trend. For example, changes in temperature and rainfall in temperate zones may favour the proliferation of certain mosquito vectors.

Therefore, in the last decade, the number of emerging flaviviruses identified worldwide has considerably increased, causing a rise in human outbreaks which represents an emerging threat to human health [3]. *Flavivirus* is a genus of positive-strand RNA viruses in the family *Flaviviridae* that are mainly transmitted by the bite of an infected arthropod (such as a mosquito or tick) and hence are classified as arboviruses. Amongst the instances which deserve special attention are the recent expansion of emerging neurotropic arthropod-borne viruses, such as West Nile virus (WNV), Usutu virus (USUV), and tick-borne encephalitis virus (TBEV), beyond their natural range of distribution, especially in Europe.

USUV and WNV are characterised by overlapping geographic distribution, host and vector species, and similar clinical manifestations. They are maintained in the environment through an enzootic cycle involving mosquitoes and birds, but they also occasionally infect humans and other mammals. Disease symptoms in vertebrates can range from mild febrile illness to severe neurological disorders (such as meningitis, encephalitis, or meningoencephalitis in humans). USUV and WNV co-circulate in parts of southern Europe, but the distribution of USUV extends into central and northwestern Europe. Few European countries implement routine surveillance programmes, and the majority of USUV and WNV infections in humans are asymptomatic, making it likely that the circulation of these viruses is underestimated. Despite this, it is now documented that USUV is rapidly spreading in several European countries and that WNV has widely spread in central and southern European countries, causing larger epidemics [4,5]. A large USUV epidemic affecting birds was reported in northern Europe, with a major circulation of the disease in Belgium, Germany, France, and the Netherlands, during the summer of 2016 [6]. Subsequently, 2018 saw both the largest USUV epizootic, causing massive mortality in several bird species in several European countries, and the largest European WNV epidemic, resulting in 2083 human cases and 181 recorded deaths [6,7]. The epidemiological situation may have recently changed in several European regions, shifting from unregular epidemics to endemicity. Despite their potential impact on human and veterinary health, limited measures are available to control these viruses. Although inactivated and recombinant WNV vaccines exist for horses [8], neither specific treatments nor vaccines currently exist for WNV or USUV for humans. Their management is thus largely based on integrated surveillance activities that support the implementation of mainly preventive measures when cases are detected.

For its part, TBEV is one of the most frequently reported tick-borne diseases in Europe. It is endemic in many European countries, including the Baltic and Scandinavian countries, Slovenia, and the Czech Republic. The rise in the number of reported cases in recent years is likely driven by several factors, including improved diagnostic tests, increased disease awareness, an increase in time spent outdoors, as well as rising temperatures. TBEV is maintained in an enzootic cycle involving hard ticks and rodents as the main reservoir [9]. It is suspected that birds play a role in the virus’s spread [10]. Thousands of TBEV neurological cases are recorded every year in Europe [11]. Human infections can occur through bites from TBEV-infected ticks, and more rarely through the consumption of unpasteurised milk or milk products from cows, goats, or sheep [12].

The control of arboviruses expansion relies mainly on surveillance and the adoption of preventive measures where cases are detected. The spatial and temporal overlap of flaviviruses poses significant challenges for surveillance and control, highlighting the need for reinforced measures of monitoring and surveillance programmes. Indeed, the current situation calls for a rigorous analysis of the epidemiological situation in Europe. This information would be essential to the design of future surveillance strategies. One of the main public health issues related to the expansion of flaviviruses is the associated neurological damage. Numerous flaviviruses, such as WNV, USUV, and TBEV, can reach the CNS and cause neuronal defects through pathogenic mechanisms which remain largely unknown. Understanding the mechanisms behind neuroinvasion and its associated neuroinflammation during arbovirus infection is urgent, as symptoms can prove severe, trigger long-term impairment, and sometimes even be fatal [13]. Therapeutical approaches towards the prevention of neurological impairments during arbovirus infections are still poorly developed.

This Special Issue entitled “Usutu, West Nile, and tick-borne encephalitis viruses” assembles a collection of original research articles and scientific reviews highlighting the critical advances in our understanding of all aspects of viral infection related to these arboviruses (or other neurotropic flaviviruses), including their cellular, molecular, and immunological aspects, alongside epidemiological, vector competence, and diagnostic studies.
